# Artificial Breath Classification Using XGBoost Algorithm for Diabetes Detection

**DOI:** 10.3390/s21124187

**Published:** 2021-06-18

**Authors:** Anna Paleczek, Dominik Grochala, Artur Rydosz

**Affiliations:** Institute of Electronics, Faculty of Computer Science, Electronics and Telecommunications, AGH University of Science and Technology, al. A. Mickiewicza 30, 30-059 Krakow, Poland; grochala@agh.edu.pl (D.G.); rydosz@agh.edu.pl (A.R.)

**Keywords:** breath acetone, diabetes, XGBoost, VOCs, machine learning, algorithms, e-nose

## Abstract

Exhaled breath analysis has become more and more popular as a supplementary tool for medical diagnosis. However, the number of variables that have to be taken into account forces researchers to develop novel algorithms for proper data interpretation. This paper presents a system for analyzing exhaled air with the use of various sensors. Breath simulations with acetone as a diabetes biomarker were performed using the proposed e-nose system. The XGBoost algorithm for diabetes detection based on artificial breath analysis is presented. The results have shown that the designed system based on the XGBoost algorithm is highly selective for acetone, even at low concentrations. Moreover, in comparison with other commonly used algorithms, it was shown that XGBoost exhibits the highest performance and recall.

## 1. Introduction

Nowadays, groups of researchers are focused on non-invasive methods for diagnosing various diseases. One of the promising tools is exhaled breath analysis. Its potential in medical diagnosis has been known since the time of Hippocrates when he used the smell of the breath to diagnose liver disease and uncontrolled diabetes [1].

**Figure 1:**
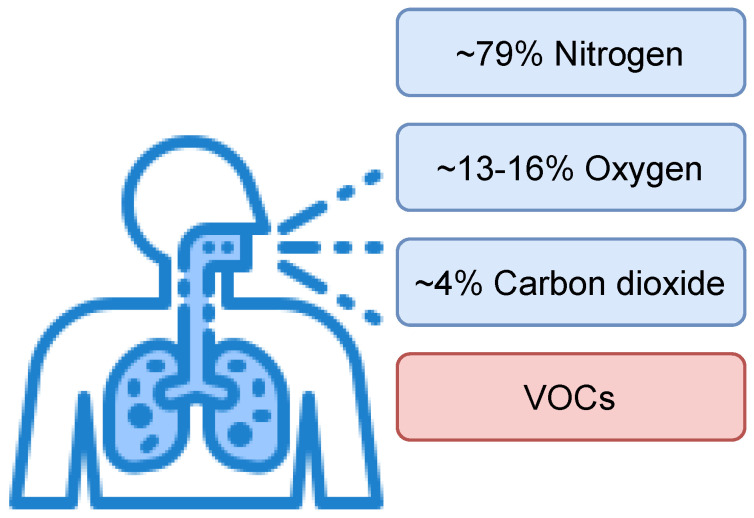
General composition of humans’ exhaled breath.

The air inhaled and exhaled by humans consists mainly of nitrogen, oxygen and carbon dioxide (Figure 1). Exhaled air contains more carbon dioxide and less oxygen than inhaled air because oxygen is used to generate energy during respiration, while carbon dioxide is produced as a by-product of the energy production process. Among the major components, exhaled breath consists of over 3500 Volatile Organic Compounds (VOCs) and a single breath consists of around 500 various VOCs, which are typically in the part per million (ppm), part per billion (ppb) or part per trillion (ppt) range [2]. Some of them are named biomarkers since their presence, as well as various concentration levels, may indicate several diseases. Biomarkers are compounds present in the body that can be used as indicators of physiology and diseases present. These types of VOCs are called endogenous VOCs and are produced by the metabolism of cells. On the other hand, the second type of VOCs are exogenous VOCs used to assess the effects of substances such as drugs, diet, cigarettes, toxic or noxious vapors and environmental pollution on the body. Exogenous VOCs are present in, for example, breath or blood as a result of circulation and/or internal metabolism [3,4,5]. Clear separation of biomarkers into these two groups is not possible because the same VOCs can be induced physiologically in the body as a result of disease, and also under the influence of external factors [4,5]. A general approach to determining biomarkers for a given pathological condition is to compare the VOC composition of a group of healthy and sick people [3]. There are several types of biomarkers: monitoring, predictive, prognostic, safety and susceptibility/risk biomarkers [6]. Systemic biomarkers are used to determine the functioning of the whole organism, while lung biomarkers are used to determine the processes and changes taking place in the respiratory system [7]. Currently, research is focused on biomarkers of various diseases, for example asthma [8,9], various types of cancers [10,11,12,13], chronic obstructive pulmonary disease [14,15] and, recently, metabolic disorders, such as diabetes [7,16,17,18,19,20,21,22,23,24], which will allow non-invasive detection and monitoring of these diseases using exhaled air. However, diet and pathological changes may affect the exhaled breath compositions; therefore, every person has their own unique molecular breath signature [7,25]. Similarly to a fingerprint, the exhaled profile is called the “breath-fingerprint” or “personal breath profile”. Common biomarkers of several diseases are listed in Table 1.

Usually, the biomarker concentrations are too low to be detected without the utilization of advanced analytical systems such as GC/MS (Gas Chromatograph coupled to a Mass Spectrometer) [37,38], SIFT–MS (Selected Ion Flow Tube–Mass Spectrometry) [39,40], PTR–MS (Proton Transfer Reaction–Mass Spectrometry) [41]. One of the promising techniques to increase the volume of biomarkers is the utilization of preconcentrators, including micropreconcentrators [22,42,43].

One disease prevalent in civilization that requires constant monitoring is diabetes. Briefly, there are two main types of diabetes: type 1 (T1DM) and type 2 (T2DM); T2DM is the most common (90% of all cases). According to data provided by the World Health Organization (WHO), approximately 500 million people worldwide have diabetes, and this number is constantly growing. The vast majority of them live in low- and middle-income countries. The WHO also reports 1.6 million deaths annually from diabetes [44]. Diabetes over time damages the nervous system, blood vessels and heart, as well as the eyes and kidneys, leading to an increased risk of premature death [45]. Due to the ever-increasing number of people with diabetes and deaths from it, the WHO reports that there is a globally agreed goal to halt the development of diabetes and obesity by 2025 [44]. At present, there are no known methods of preventing type 1 diabetes. Its treatment consists of continuous monitoring of blood glucose level (BGL) and the patient’s insulin intake. However, in the case of type 2 diabetes, it is possible to reduce its incidence by adhering to a proper diet, increasing physical activity, and reducing smoking. In addition to diet and exercise, early diagnosis plays an important role in the treatment of diabetes, so it is important to develop an easily accessible and non-invasive device that can be used for screening [44,45,46]. In terms of exhaled breath analysis, acetone was identified as a biomarker of diabetes [7,16,17,18,19,20,21,22,23,24,47]. Results presented in Table 2 show that breath acetone concentrations for healthy peoples were lower than for diabetes patients.

Experimental results have shown that relative humidity (RH) and temperature of exhaled human breath vary between subjects. Mansour et al. examined Parisian and Halifa participants. The measured values were 31.4–35.4 °C and 65.0–88.6% for Halifa participants and 31.4–34.8 °C and 41.9–91.0% for Parisian participants [52]. Ferrus et al. showed that the RH in exhaled air from humans varies between 89 and 97% [53]. Due to the high relative humidity of the breath and its influence on the sensitivity of the measurement systems (especially metal oxide semiconductor sensors) [54,55,56], it is necessary to use moisture absorbers to properly store the breath samples and to take into account the influence of humidity on the measurements in designed algorithms.

The researchers present the results of using various supervised machine learning and deep learning algorithms to classify breath samples and detect diabetes. The most popular are K Nearest Neighbours (KNN) [57,58,59,60], Support Vector Machines (SVM) [37,59,61,62,63], Naive Bayes (NB) [59,64], Deep Neural Network (DNN) [59] and also Convolutional Neural Networks (CNN) [65]. The extraction and selection of features was most often performed using Principal Component Analysis [57,59,61,66]. The main limitation of the conducted research is the lack of an adequate number of patient samples. Only a small fraction of the research has been carried out on sample numbers above a hundred [57,58,61].

In this paper, the experimental results on the e-nose system for discrimination between healthy and diabetic patients based on the exhaled breath analysis are presented. Within this study, an artificial breath profile was developed to simulate real conditions and enable testing without involving real samples.

## 2. Materials and Methods

**Figure 2:**
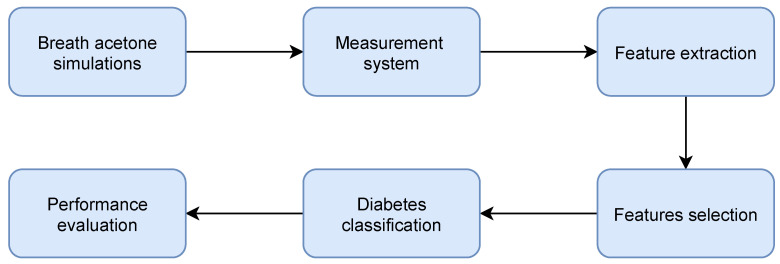
Block scheme of the proposed system.

The scheme of the system proposed in this paper is presented in Figure 2.

All algorithms were developed using scikit-learn Machine Learning in Python [67,68] and XGBoost, an open-source software library that provides a gradient boosting framework for C++, Java, Python, R, Julia, Perl, and Scala [69].

### 2.1. Equipment

**Figure 3:**
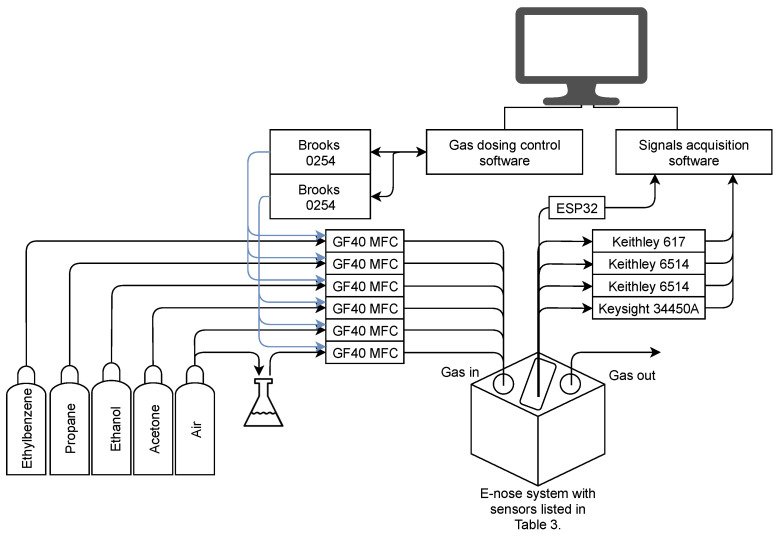
Scheme of the proposed measurement system.

Selected gas sensors (listed in Table 3) were placed in a measurement chamber with a 180 mL capacity and supplied with appropriate voltages in accordance with their data sheets. Due to the relative humidity influence on sensors’ sensitivity, in addition to gas sensors, temperature, relative humidity and pressure sensors were also used. The BME280 (Bosch Sensortec, Reutlingen, Germany) and SHT85 (Sensirion, Staefa ZH, Switzerland) sensors were placed inside the measurement chamber, while the second SHT85 sensor was placed before the gases entered the measurement chamber. All used sensors, except SGP30 and SHT85, responded to the dosed gases as voltage. For SGP30, the sensor returned Total Volatile Organic Compounds (TVOCs) and an equivalent carbon dioxide reading (eCO2) over the I2C communication bus. TGS1820 (Figaro Engineering Inc, Mino, Osaka, Japan), TGS2620 (Figaro Engineering Inc, Mino, Osaka, Japan), TGS8100 (Figaro Engineering Inc, Mino, Osaka, Japan), MQ3 (Waveshare, Shenzhen, China) and MICS5524 (Amphenol SGX Sensortech, Corcelles-Cormondreche, Switzerland) sensors’ responses were measured using Keithley 617 (Tektronix, Beaverton, OR, USA), Keithley 6514 (Tektronix, Beaverton, United States) and multimeter Keysight 34450A electrometers (Keysight, Santa Rosa, CA, USA). If the sensor sent the measured values using the Serial Peripheral Interface (SPI) or Inter-Integrated Circuit (I2C) communication bus, the ESP32 dev board (Espressif Systems, Shanghai, China) was used to read these values and send them to the measurement application written in the Python programming language. Figure 3 shows a scheme of the proposed e-nose measurement system. The glass flask shown in Figure 3 was used to simulate the humidity.

### 2.2. Exhaled Breath Simulations

The gas mixtures composed of synthetic air, acetone, ethanol, propane and ethylbenzene were dosed with a variable relative humidity to simulate exhaled air using the GF40 series (Brooks, Hatfield, United States) mass flow controllers with a Brooks 0254 controller. Due to the high humidity of the exhaled air, the measurements simulated humidity ranging from 0 to 70%. However, the relative humidity measured inside the chamber was 0 to 40% due to the increased temperature in the measurement chamber. Taking into account the number of all possible combinations of gas mixtures, the total duration of measurements was estimated to be more than 700 days. Thanks to the use of an artificial exhaled breath mixture, the experiments could be conducted constantly (24 h/7 d) without involving the diabetic patients. Since acetone is the key biomarker of diabetes, it was decided to measure the response to various concentrations of acetone contaminated with other gases in the concentration ranges that have been previously confirmed by the utilization of analytical techniques such as GC/MS [37,38]. Based on the obtained results presented in Table 2, the simulations assumed that the concentration of acetone in the exhaled air for a healthy person is <1.5 ppm and for a diabetic patient is ≥1.5 ppm.

### 2.3. Preprocessing

**Figure 4:**
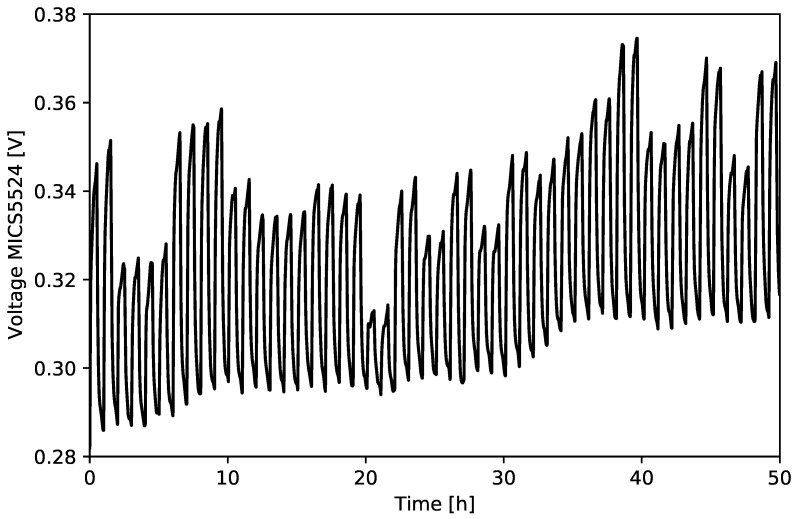
Result of the long-term test for different gas mixtures—MICS5524.

In order to obtain input data for the algorithms, preprocessing and features extraction were carried out. The use of baseline subtraction is important due to baseline drift. The result of the long-term stability test is given in Figure 4.

**Figure 5:**
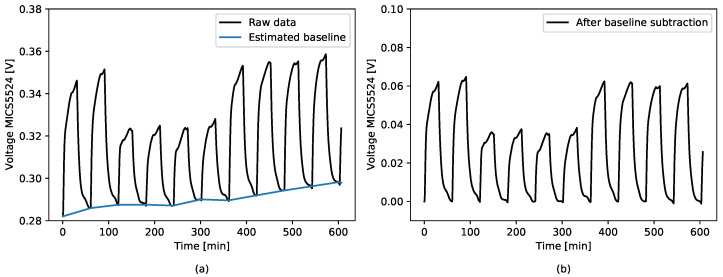
Baseline subtraction. (**a**) Sensor raw response with fitted baseline; (**b**) result of the baseline subtraction.

The baseline was fitted to the raw data obtained from the sensors and then subtracted (Figure 5).

The following features have been extracted from each gas sensor:

- The sensor response (S) defined by Equation (1):
  (1) $$S=\frac{R_{S}}{R_{0}}$$
- The sensor response change (ΔS) defined by the Equation (2):
  (2) $$\Delta S=R_{S}-R_{0}$$
  where:
  $R_{S}$—sensor exposed to target gas, e.g., acetone;
  $R_{0}$—sensor exposed to pure synthetic air;
- Area under sensor’s response curve (AUC) calculated when the sensor is exposed to gas. Result approximated by the trapezoidal numerical integration.

**Figure 6:**
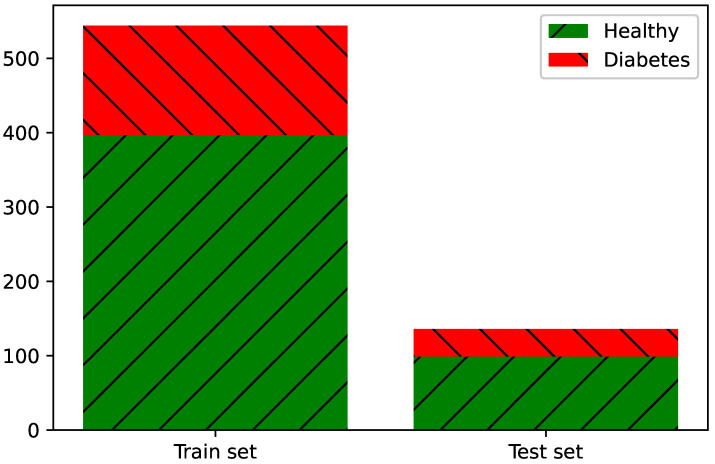
Dataset abundance and distribution.

The prepared dataset from the simulation of acetone in the breath was divided into two separate sets—the training set and the test set. In order to simulate the real case, where samples from healthy subjects are overwhelmingly obtained [37,38,70,71], the simulations were conducted with an unbalanced number of samples. Moreover, not every algorithm, i.e., Support Vector Machines, K Nearest Neighbours [72,73,74], works well with an unbalanced dataset; therefore, such experiments are crucial. Due to the unbalanced number of samples belonging to the “healthy” and “diabetes” classes, the data were divided in such a way that the same percentage of samples from each class was included in both the test and training sets. Distribution of samples in the dataset are given in Figure 6.

### 2.4. Features Selection

Due to the correlation between the features extracted from the raw data from each sensor, we decided to use the calculated *S* results and the values read from the temperature and humidity sensors as an input to the algorithms. As detailed in Section 3.3, the gas sensors, except SGP30, used the *S* value that slightly changes with the change in humidity, which is important when measuring exhaled air, characterized by high humidity.

### 2.5. XGBoost Classifier

Recently, extreme gradient boosting (XGBoost) state-of-the-art algorithms are becoming more and more popular not only for classification, but also for regression problems, due to their high performance [69,75,76,77]. The XGBoost alghorithm is a scalable tree boosting system which was developed by Chen and Guestrin in 2016. Parallel, distributed, out-of-core and cache-aware computing makes the algorithm more than ten times faster than popular models used in machine learning (ML) and deep learning (DL). Another advantage of this algorithm is that it is well optimized and scalable. Due to this innovation, it can be successfully used to process billions of examples in distributed or memory-limited settings. This cutting-edge application of gradient boosting machines was designed to handle real-world problems where the input data sparsity is a common issue. The algorithm is aware of the presence of missing values, too frequent zero values in the dataset and results of applied feature engineering techniques. The ensemble technique is the recursive addition of new models until further addition no longer noticeably enhances the performance of existing models. The loss of the model is minimized by the gradient descent algorithm [69].

### 2.6. Hyperparameter Optimization

To determine the best performance, the model’s hyperparemeters were optimized by a grid search algorithm. Model evaluation was performed using the stratified k-fold cross-validation method. It is commonly used to evaluate models with limited datasets. We decided to use a stratified version of this algorithm due to the unbalanced dataset; it splits the dataset, keeping the equal proportions of each output class in each fold. The use of this method enables the selection of optimal model hyperparameters and reduces overfitting of the data. The training set was divided into *k* sets, then the model was trained with the use of *k*−1 datasets, and the remaining set was used to validate the model using the selected metrics. The final value of a metric is the average of the *k* iteration [78,79].

### 2.7. Classifiers’ Performance Evaluation Metrics

In this paper, we mainly focused on obtaining the highest possible sensitivity value (recall score) defined by Equation (3):

(3) $$TPR=\frac{TP}{TP+FN}$$

where:

*TPR*—true positive rate (recall, sensitivity);

*TP*—true positive;

*FN*—false negative [80].

This metric is especially important in medical applications, when the dataset is unbalanced, and we strive to minimize the type II error. For example, in the case of screening tests, it is important to mark all potentially sick patients and possibly, in further, more accurate, as well as invasive and more expensive tests, confirm or rule out diabetes.

## 3. Results and Discussion

### 3.1. Sensors’ Sensitivity to Gases Used in Simulations

**Figure 7:**
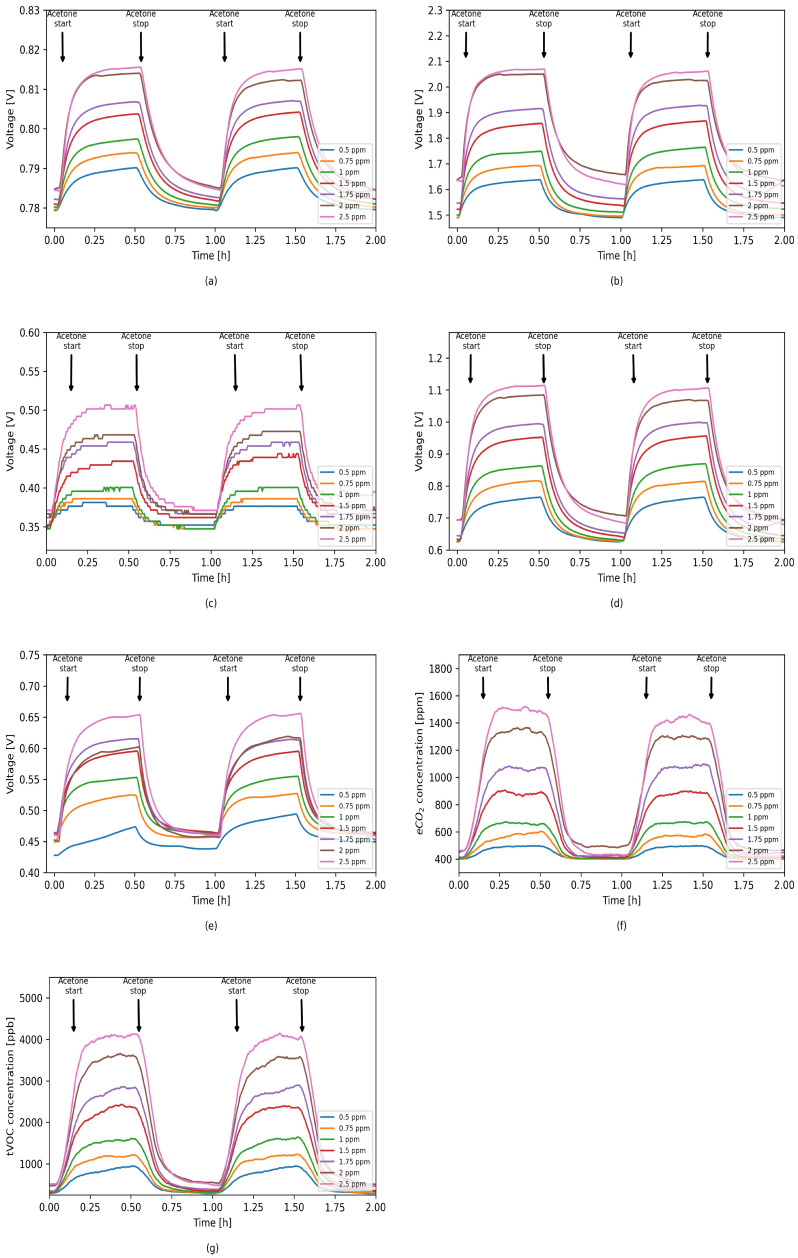
Sensors’ responses to different acetone concentrations in 0% RH. (**a**) TGS1820; (**b**) TGS2620; (**c**) TGS8100; (**d**) MQ3; (**e**) MICS5524; (**f**) SGP30 eCO2; (**g**) SGP30 tVOC.

Figure 7 shows the responses of each sensor to different acetone concentrations. Each concentration was repeated at least twice in order to check the stability of the sensors and the repeatability of the response to individual gas concentrations. The results show that each of the sensors is sensitive to changes in acetone concentration, and in the case of the same concentration being used several times, the sensors are stable and the responses are repeatable.

### 3.2. Sensors’ Selectivity to Acetone

**Figure 8:**
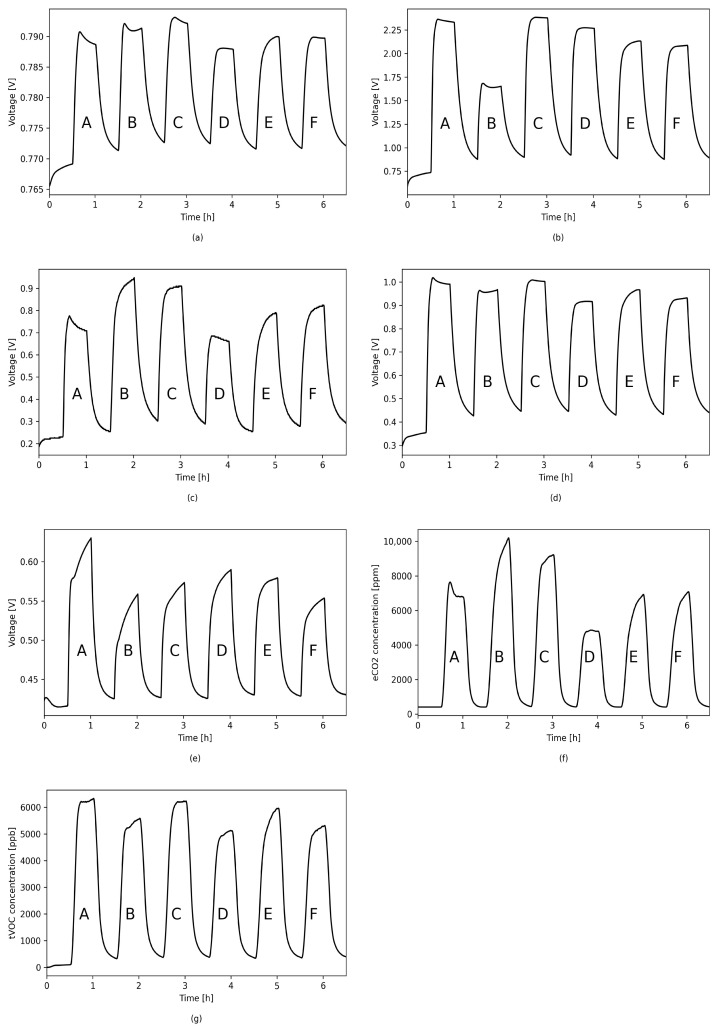
Sensors’ responses to different simulated mixtures in 0% RH. A. 1.5 ppm acetone, 2.5 ppm ethanol, 1 ppm propane; B. 1.5 ppm acetone, 1 ppm ethanol, 2.5 ppm ethylbenzene; C. 1.5 ppm acetone, 1.5 ppm ethanol, 1 ppm ethylbenzene, 1 ppm propane; D. 1.5 ppm acetone, 1.5 ppm ethanol, 1 ppm propane; E. 1.5 ppm acetone, 1.5 ppm ethanol, 0.5 ppm ethylbenzene, 0.5 ppm propane; F. 1.5 ppm acetone, 1 ppm ethanol, 1 ppm ethylbenzene, 0.5 ppm propane; (**a**) TGS1820; (**b**) TGS2620; (**c**) TGS8100; (**d**) MQ3; (**e**) MICS5524; (**f**) SGP30 eCO2; (**g**) SGP30 tVOC.

The results of measurements of the sensor response to various gas mixtures with a constant concentration of acetone—1.5 ppm in each mixture, given in Figure 8—show that none of the sensors included in the designed e-nose system is fully acetone selective. Therefore, it is important to use a sensor array where each sensor is selective for different gases/gas mixtures.

### 3.3. Relative Humidity Dependency

**Figure 9:**
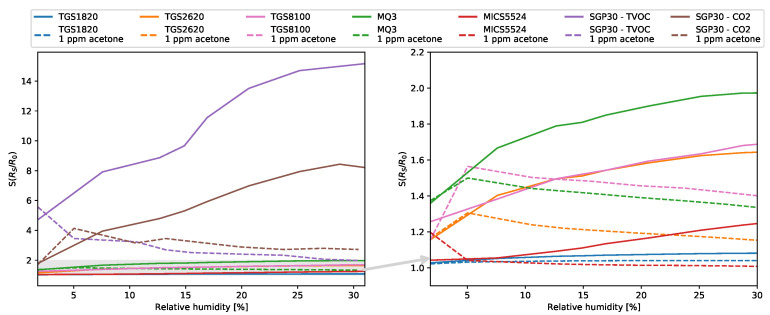
Sensors’ sensitivity in different relative humidities in chamber.

Due to the high humidity of the breath, measurements were made at different simulated humidities. For each of the sensors used, the characteristics of the relative dependence of the sensor’s response to humidity were determined and the dependence of the sensitivity to 1 ppm of acetone on the ambient humidity was also calculated. Results are given in Figure 9.

### 3.4. Classification

**Figure 10:**
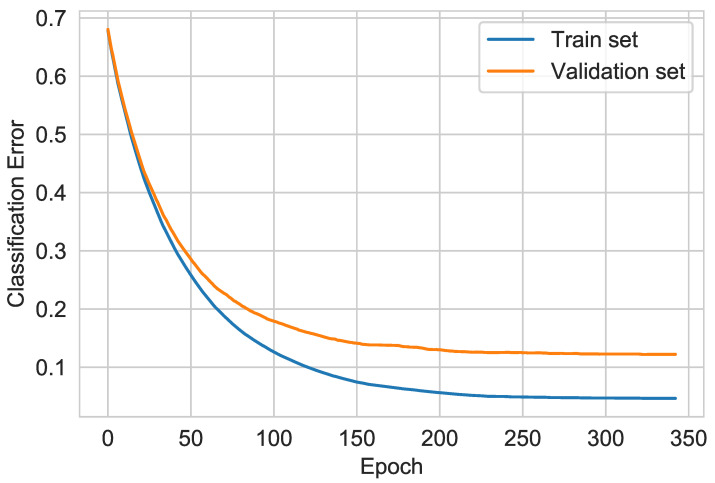
XGBoost learning curves.

The optimal model hyperparameters were determined using the grid search algorithm. In order to assess whether the model is underfitted or overfitted, validation was used with the use of a separate validation set. Learning curves showing the dependence of the classification error on the number of training epochs are shown in Figure 10.

### 3.5. Feature Importance

**Figure 11:**
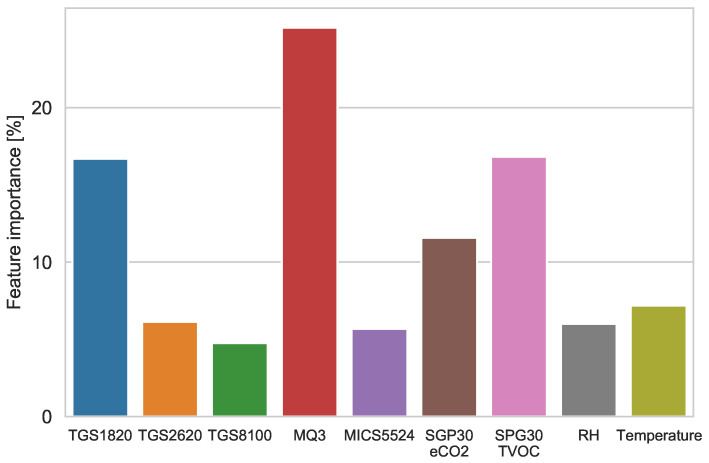
XGBoost features’ importance.

The results of the algorithm showed that the three most important features for the classification were measurements from the MQ3, TGS1820, SGP30 and SHT85 sensors placed inside the chamber. Feature importance values for the most significant sensors are given in Figure 11.

### 3.6. Performance Evaluation

In the case of using the algorithm based on the gradient of boosted trees, the recall equals 1, which means that all the sick patients were correctly marked as sick and the type II error was minimized. The other calculated performance evaluation metrics are summarized in Table 4. As we assumed, the algorithm’s hyperparameters were selected in such a way that it achieved the highest recall value.

#### Confusion Matrix

**Figure 12:**
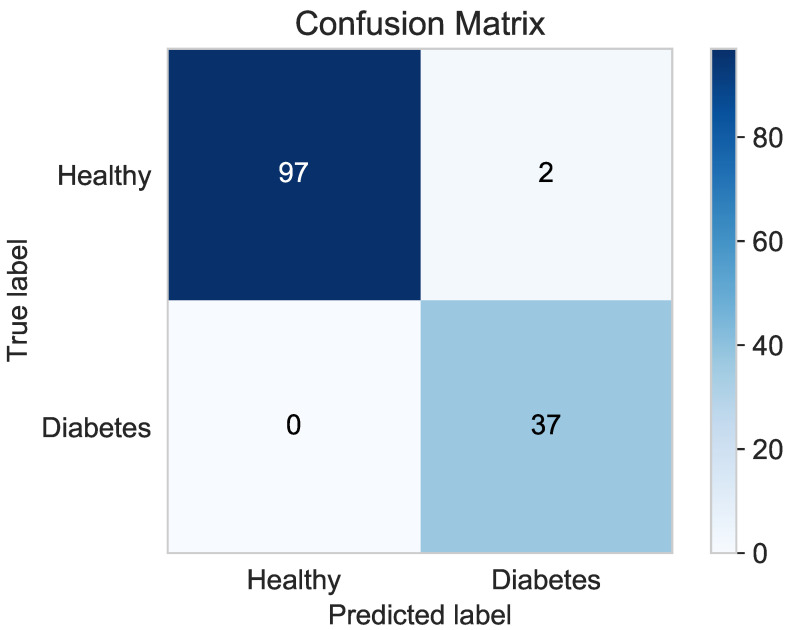
XGBoost Classifier confusion matrix.

The algorithm’s confusion matrix is shown in Figure 12. It shows that the healthy diabetes samples were classified properly. The confusion matrix allows one to accurately quantify the true positive, true negative, false positive and false negative test samples. Based on these values, the remaining metrics are calculated. In the case of the proposed XGBoost Classifier algorithm, two cases of simulated diabetes patients were incorrectly classified. This is a type I statistical error.

### 3.7. Comparison with Classic Machine Learning Algorithms

In this paper, we also compared the classification performance achieved using the XGBoost algorithm with the results of classic classifiers such as Support Vector Machines (SVM), K Nearest Neightbour (KNN), Decision Tree Classifier (DT) and Random Forest Classifier (RF), commonly used in previous research. For these algorithms, the hyperparameters were also determined using the grid search method and the K-Fold validation was performed. The classification was carried out using the same train and test sets as for XGBoost.

**Figure 13:**
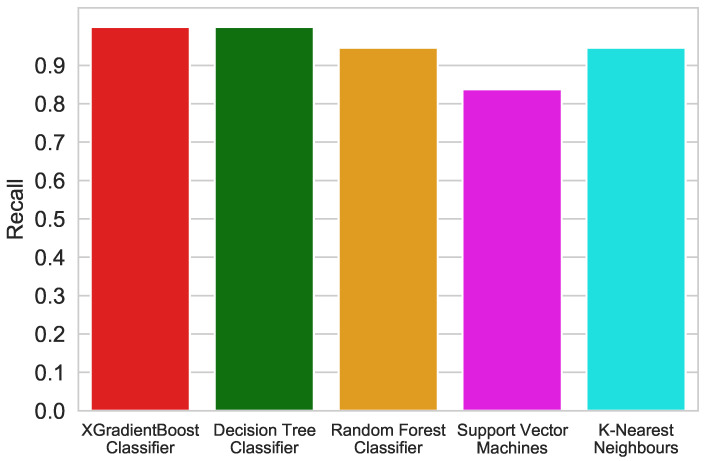
Recall comparison of different algorithms.

Figure 13 shows a comparison of the achieved recall of the algorithms.

**Figure 14:**
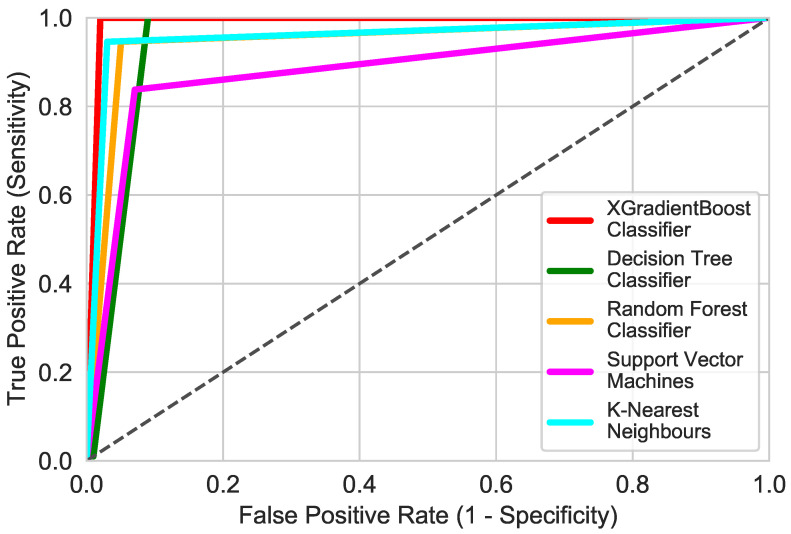
ROC comparison of different algorithms.

The receiver operating characteristics (ROC) curve shows the dependence between recall and 1-specificity. It is commonly used in machine learning tasks for medical applications. The closer the curve for a given model is to the point (0,1), the better the classifier. The most common problem in designing models for medical data is that the data contain more healthy cases than disease ones [81]. Figure 14 shows the ROC comparison for each of the algorithms used in this research.

All of the used algorithms exhibited good performances. Each of these algorithms obtained recall and false positive rates of over 80%. By analyzing the determined metrics, it can be seen that the XGBoost Classifier has the highest accuracy and recall equal to 99 and 100%, respectively. Decision Tree Classifier obtained a recall identical to the XGBoost Classiffier, but the results differ in the amount of false positives. It is true that in screening tests, the most important detection is as many true positives as possible, but reducing the number of false positives, i.e., healthy ones classified as sick, reduces the cost of further diagnosis.

### 3.8. Discussion

Due to the individual variability shown in the literature, depending on, inter alia, sex, age, diet, duration of diabetes life, the course of treatment and its type, it is necessary to conduct tests on breath samples. It may also be necessary to develop a method for calibrating the device tailored to an individual patient. The results presented in this paper show that the designed system is highly selective for acetone, even at low concentrations. In order to confirm the selectivity of the system towards all breath components, it is necessary to carry out measurements on samples of exhaled air taken from healthy people and diabetics. The graphs of dependence of the sensor’s response and sensitivity on the ambient humidity in the measurement chamber showed that the all sensors used, except SGP30, are slightly sensitive to humidity. Measurements of humidity in the chamber and taking these results into account in the input data to the algorithms made it possible to compensate for its influence. In the case of the presented sensors’ system and the algorithm used, the classification of diabetics was independent of the relative humidity inside the measuring chamber. Comparison with other commonly used algorithms showed that XGBoost showed the highest performance and recall. One of the disadvantages of the system is the long response and retention time of each of the sensors used; therefore, in order to use such a system for medical applications, it is necessary to use a different sensor matrix, a preconcentrator, increase the total air flows in the chamber or reduce the volume of the measurement chamber.

## 4. Conclusions

Exhaled breath analysis consists of several steps including sample collection, compound detection, data analysis, and data interpretation. Each stage could be realized in various manners. So far, the researchers have made efforts to develop the compound detection units, for example, by the utilization of electronic noses, which offer cheap, fast, and reliable results. However, due to the number of compounds present in exhaled human breath as well as high humidity concentration, the detection unit has to be supported by an artificial intelligence element to deliver reliable results. In this paper, the XGBoost algorithm for diabetes detection based on the exhaled breath analysis is presented. The results have shown that the designed system based on the XGBoost algorithm was highly selective for acetone, even at low concentrations. Moreover, in comparison with other commonly used algorithms, it was shown that XGBoost exhibits the highest performance and recall, which makes it a first choice for data analysis in terms of diabetes detection.

## Figures and Tables

**Table 1:** Potential disease biomarkers in the breath.

Disease	Biomarkers	References
Diabetes	Acetone	[2,7,16,17,18,19,20,21,22,23,24,26]
Asthma	Nitric Oxide	[2,8,9]
Cystic fibrosis	Hydrogen cyanide	[27,28]
Lung cancer	VOC pattern	[10,11,26]
Chronic kidney disease	Trimethylamine	[29]
Colorectal cancer	Methane	[30,31]
Myocardial infarction	Pentane	[32,33]
Obstructive sleep apnea	Pentane and Nitric Oxide	[34]
Renal failure	Ammonia	[35,36]

**Table 2:** Acetone concentration in health and diabetes samples.

Diabetic Stage	Measured Acetone Concentration	References
T2DM	1.76–3.73 ppm	[18]
Healthy	0.22–0.80 ppm	
Controlled diabetic	0.19–0.66 ppmv	[22]
Untreated T2DM	0.92–1.20 ppmv	
Diabetes	1.25–2.5 ppm (or up to 25 ppm)	[23]
Healthy	0.2–1.8 ppm	
T1DM	4.9 ± 16 ppm	[47]
T2DM	1.5 ± 1.3 ppm	
Healthy	1.1 ± 0.5 ppm	
Diabetes	>1.8 ppmv	[48]
Healthy	<0.8 ppmv	
T1DM	2.19 ppmv (mean)	[49]
Healthy	0.48 ppmv (mean)	
Healthy	0.177–2.441 ppm	[50]
Healthy	0.176–0.518 ppm	[51]

**Table 3:** Sensors used in measurements.

Sensor	Target Gases	Typical Detection Range
TGS1820	(CH${}_{3}$)${}_{2}$CO	1–20 ppm (CH${}_{3}$)${}_{2}$CO
TGS2620	C${}_{2}$H${}_{5}$OH,	50–5000 ppm C${}_{2}$H${}_{5}$OH
	Solvent apors	
TGS8100	Air contaminants	1–30 ppm H${}_{2}$
	(H${}_{2}$, C${}_{2}$H${}_{5}$OH etc.)
MICS5524	CO, VOCs	1–1000 ppm CO
		10–500 ppm C${}_{2}$H${}_{5}$OH
		1–1000 ppm H${}_{2}$
		1–500 ppm NH${}_{3}$
		>1000 ppm CH${}_{4}$
MQ3	C${}_{2}$H${}_{5}$OH, CH${}_{4}$,	0.04–4 mg/L C${}_{2}$H${}_{5}$OH
	Benzine, Hexane,
	LPG, CO
SGP30	CO${}_{2}$, VOCs	0–1000 ppm H${}_{2}$
		0–1000 ppm C${}_{2}$H${}_{5}$OH
		0–60,000 ppb eq tVOCs
		400–60,000 ppm eq CO${}_{2}$

**Table 4:** Classifier performance evaluation results.

Metric	Result
Accuracy	99%
Recall	100%
Specificity	97.9%
Area under ROC curve	97.9%
F1-score	97.4%

## Data Availability

Not applicable.
